# Recent Arctic tundra fire initiates widespread thermokarst development

**DOI:** 10.1038/srep15865

**Published:** 2015-10-29

**Authors:** Benjamin M. Jones, Guido Grosse, Christopher D. Arp, Eric Miller, Lin Liu, Daniel J. Hayes, Christopher F. Larsen

**Affiliations:** 1 Alaska Science Center, U.S. Geological Survey, Anchorage, AK 99508, USA; 2 Alfred Wegener Institute, Helmholtz Centre for Polar and Marine Research, Potsdam, Germany; 3 Water and Environmental Research Center, University of Alaska Fairbanks, Fairbanks, AK 99775, USA; 4 Bureau of Land Management Alaska Fire Service, Fort Wainwright, AK 99703, USA; 5 Earth System Science Programme, Faculty of Science, Chinese University of Hong Kong, Hong Kong, China; 6 Environmental Sciences Division, Oak Ridge National Laboratory, Oak Ridge, TN 37831 USA; 7 Geophysical Institute, University of Alaska Fairbanks, Fairbanks, AK 99775 USA

## Abstract

Fire-induced permafrost degradation is well documented in boreal forests, but the role of fires in initiating thermokarst development in Arctic tundra is less well understood. Here we show that Arctic tundra fires may induce widespread thaw subsidence of permafrost terrain in the first seven years following the disturbance. Quantitative analysis of airborne LiDAR data acquired two and seven years post-fire, detected permafrost thaw subsidence across 34% of the burned tundra area studied, compared to less than 1% in similar undisturbed, ice-rich tundra terrain units. The variability in thermokarst development appears to be influenced by the interaction of tundra fire burn severity and near-surface, ground-ice content. Subsidence was greatest in severely burned, ice-rich upland terrain (yedoma), accounting for ~50% of the detected subsidence, despite representing only 30% of the fire disturbed study area. Microtopography increased by 340% in this terrain unit as a result of ice wedge degradation. Increases in the frequency, magnitude, and severity of tundra fires will contribute to future thermokarst development and associated landscape change in Arctic tundra regions.

Wildfire disturbance in northern high latitude ecosystems is an important factor contributing to permafrost degradation^1^,^2^,^3^. Fire-induced permafrost degradation is well documented across the boreal forest region^4^,^5^,^6^,^7^,^8^ where ground temperatures are relatively warm and permafrost is discontinuous^9^. Severe burning removes the vegetation and overlying surface soil organic matter and the loss of this insulating layer causes ground temperatures to warm and the thickness of the seasonally thawed active layer to increase^7^,^10^,^11^. In the boreal forest region, complete degradation of near-surface permafrost may occur 3–5 years following a severe burn^7^,^12^. In ice-rich permafrost terrain, the melting of ground ice causes surface subsidence, leading to the formation of thermokarst and other thaw-related landforms^1^,^4^,^7^,^13^. The impact of fires on cold, continuous permafrost-influenced tundra terrain is less well understood^14^,^15^,^16^. While several studies have documented changes in active layer thickness following tundra fire^17^,^18^,^19^, few studies have reported on thermokarst development in response to Arctic tundra fire disturbances^14^,^20^,^21^. In both Boreal and Arctic permafrost regions, detailed analyses of the landscape-scale impacts of fire on permafrost degradation and potential development of thermokarst terrain is currently lacking.

Better understanding the processes controlling thermokarst initiation in Arctic and Boreal regions is important since they contain globally significant amounts of carbon^22^, primarily stored in permafrost soils^23^ and peatlands^24^. Fire in these regions act as a pulse disturbance mechanism that mobilizes carbon through combustion of vegetation^25^,^26^,^27^,^28^,^29^ and burning of surface soil organic layers^30^,^31^, and may result in the additional release of soil organic carbon through post-fire permafrost degradation^8^,^11^,^32^. Fire is considered one of the primary disturbance mechanisms in boreal forests^27^,^33^. Recent shifts in the boreal forest fire regime have resulted in carbon emissions that exceed decadal-scale carbon storage during the past 60 years^29^,^34^. Such shifts also substantially influence vegetation composition^35^, land-atmosphere energy exchange^33^, the soil thermal regime^7^,^36^,^37^, and future fire dynamics^38^. While fires in Arctic tundra have been comparatively infrequent during the past 60 years, limited data indicate an increase in their occurrence over decadal^19^ and millennial^39^,^40^ time-scales. The potential for a new disturbance regime in a warming Arctic highlights the need to better understand the landscape-scale drivers and impacts of fire on permafrost.

Remote sensing provides a means for documenting and quantifying many of the various changes occurring across Arctic landscapes in recent decades^41^. In 2007, the Anaktuvuk River tundra fire burned ~1,000 km^2^ in northern Alaska (Fig. 1). Analysis of multi-resolution spaceborne optical data showed that ~50% of the area burned at high severity^42^,^43^. In this severely burned tundra, the fire burned the surface vegetation layer and consumed nearly 30 cm of the insulating surface soil organic layer, often down to mineral soil^15^,^31^. Spaceborne Interferometric Synthetic Aperture Radar (InSAR) data was recently used to analyze whether this severe tundra burning resulted in vertical land surface deformation change post-fire^21^. The time series indicated a 2 to 8 cm increase in thaw season surface subsidence between 2006 (pre-fire) and 2010 (post-fire) that was attributed to a combination of both active layer thickening and permafrost thaw subsidence following the fire^21^. However, interpretation of terrain subsidence remained inconclusive due to uncertainties associated with the InSAR techniques, the loss of image coherence across large areas within the burn, the influence of surface soil organic layer combustion on detected subsidence, and the relatively coarse spatial resolution (>10 m) of the data.

In this study, we investigate the impact of the Anaktuvuk River tundra fire on potential, post-fire thermokarst development and how this might vary given differences in burn severity and inferred ground-ice content at the landscape-scale. To address these questions, we used two airborne LiDAR datasets, acquired two and seven years following the large and severe Anaktuvuk River tundra fire, to quantify the landscape scale impacts of the fire on Arctic permafrost terrain. The first LiDAR dataset was acquired in July 2009, covering 650 km^2^ of the burn area. A second LiDAR dataset was acquired in July 2014, overlapping 350 km^2^ of the 2009 dataset, with 310 km^2^ located within the burn perimeter and 40 km^2^ located outside of the burn perimeter. Digital terrain models (DTMs) at 1 m spatial resolution were developed for each acquisition, and differenced to assess thermokarst development between the two datasets. Landscape-scale changes in the differential digital terrain models (dDTMs) were analyzed according to terrain units that represent a gradient in geomorphic character and the amount and distribution of ground ice in the study area^44^. A Landsat-derived burn severity metric was used to further analyze spatial differences in the response of the landscape to the tundra fire event. To our knowledge, this is the first study to demonstrate the utility of multi-temporal airborne LiDAR data for documenting landscape-scale, fire-induced thermokarst terrain formation in the Arctic or Boreal region. Our results indicate that the impact of tundra fires for initiating widespread thermokarst development in regions with ice-rich permafrost in the Arctic has been underestimated.

## Results

### Thermokarst development post-fire

**Figure 2:**
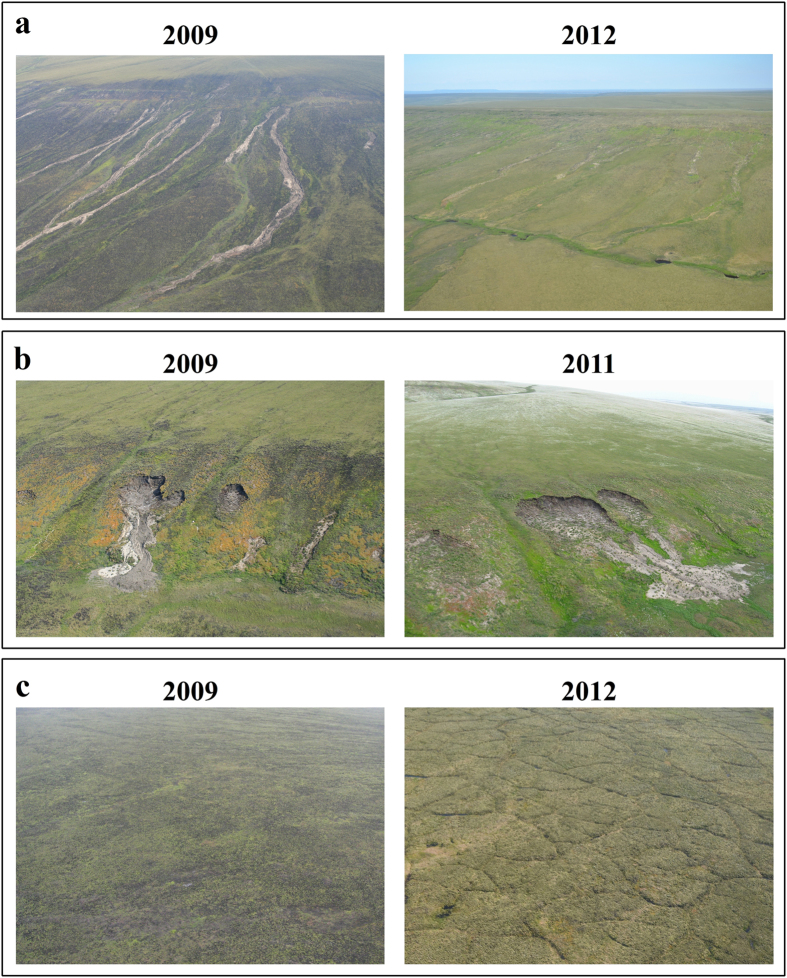
Rapid (**a,b**) and delayed (**c**) thermokarst development following the Anaktuvuk River tundra fire. (**a**) Active layer detachment slides occurred in the first few years following the fire (2009) but were largely stabilized five years post-fire (2012). (**b**) Retrogressive thaw slumps were also triggered immediately following the fire (2009). They have expanded locally but remained relatively uncommon and have begun to stabilize four years post-fire (2011). (**c**) Widespread ice-wedge degradation was not evident in the first few years following the fire (2010) but noticeable following the fifth year post-fire (2012). Image pairs show the same location but from slightly different perspectives and in different years. The location of these sites are shown in Fig. 1. Image credits: BLM Alaska Fire Service (a-2009, b-2009, b-2011) and BMJ (a-2012, c-2010, c-2012).

**Figure 3:**
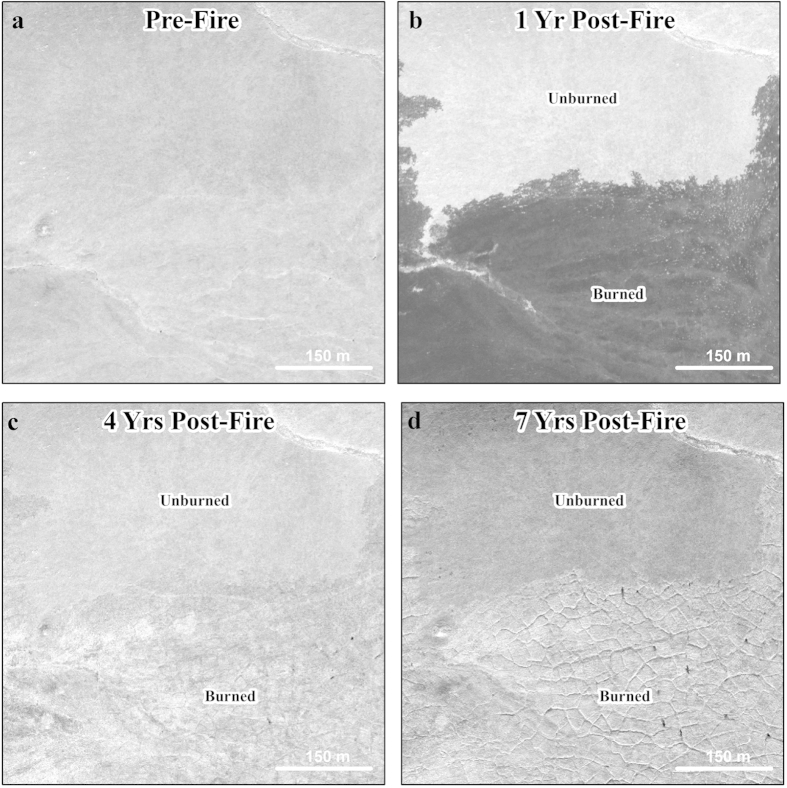
Time series of high-resolution (<1 m) satellite imagery (copyright DigitalGlobe, Inc.) showing ice wedge degradation associated with burned upland (yedoma) tundra. (**a**) A Quickbird image from 27 June 2006, the year prior to the Anaktuvuk River fire. (**b**) A Quickbird image from 05 July 2008, the year following the fire, showing a portion of the northern extent of the burn area. (**c**) A Worldview-1 image from 02 July 2011, four years following the fire, showing subtle, initial signs of ice wedge degradation. (**d**) A Worldview-1 image from 06 September 2014, seven years following the fire, showing widespread ice wedge degradation in the burned area. All panels show the same location and the imagery indicates that ice wedge degradation became ubiquitous after 2011 (4 yrs post-fire). Figure created in Esri® ArcMap™ 10.1.

The development of thaw-related landforms was observed in the field during the first two summers following the Anaktuvuk River tundra fire (Fig. 2). Both active layer detachment slides (ALDS) and retrogressive thaw slumps (RTS) were triggered shortly after the fire, but remained local in extent and largely stabilized within 5 years. In contrast, extensive subsidence associated with ice wedge degradation was not readily apparent in the field until the fifth summer (2012) following the fire (Fig. 2). Visual analysis of very high resolution (1 m) satellite imagery (Fig. 3) indicated only very subtle differences in image texture between 2008 and 2011, but between 2011 and 2014 ice wedge degradation was observed to be ubiquitous within the burn area.

**Figure 4:**
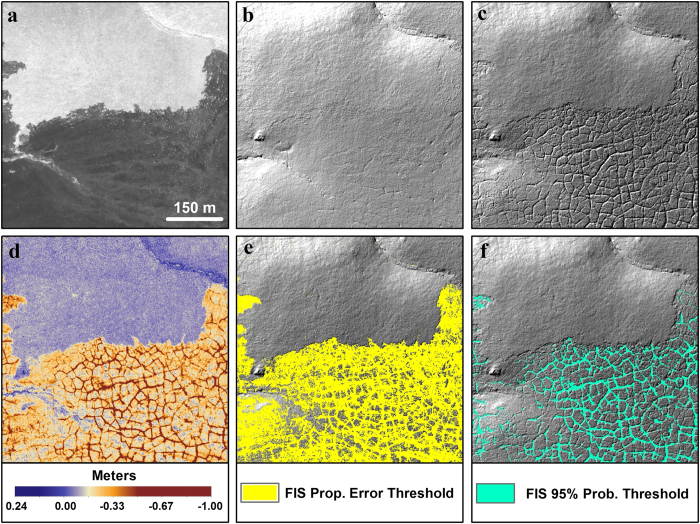
Detection of permafrost thaw subsidence and thermokarst initiation in burned tundra using multi-temporal LiDAR. (**a**) A Quickbird image from 05 July 2008, the year following the fire, showing a portion of the northern extent of the burn area and the distinction between burned (dark) and unburned (light) tundra. Hillshade images of the (**b**) 2009 and (**c**) 2014 1 m resolution LiDAR DTMs showing ice wedge degradation in the burn area. (**d**) The raw dDTM created by subtracting the 2009 DTM from the 2014 DTM. Detectable change determined using the (**e**) FIS propagation of errors threshold (>~0.2 m) and (**f**) the FIS 95% probability threshold (>~0.5 m)^45^,^53^. These panels show the same area as shown in Fig. 3. Figure created in Esri® ArcMap™ 10.1.

To analyze terrain subsidence quantitatively, we differenced 1 m resolution LiDAR-derived DTMs based on data acquired in 2009 and 2014 using the Geomorphic Change Detection software^45^ (Fig. 4; see methods section). Detectable subsidence was measured across 34% (103 km^2^) of the burned portion of the study area (Table 1). In contrast, only 5% of the study area outside of the burn perimeter was quantified as detectable subsidence, with 80% of this detected subsidence associated with changes occurring in the Itkillik River floodplain, likely reflecting mechanical erosion of river channel banks and not directly related to permafrost thaw subsidence.

We created a terrain unit classification (see methods section) in order to better understand the spatial variability of subsidence occurring over the study period and the vulnerability of different geomorphic settings to permafrost degradation post-fire. The study area was divided into seven terrain units based on the terrain unit classification and existing surficial geology maps^44^: (1) drained lake basins, (2) yedoma uplands, (3) rocky uplands, (4) previously glaciated uplands, (5) river floodplain, (6) tundra stream gulches, and (7) lakes (Table 1). These units were further distinguished on the basis of being inside or outside the burn perimeter. Drained lake basins and yedoma uplands both accounted for ~30% of the entire study area and both ~30% of the burned portion of the study area. Rocky uplands and glaciated uplands contributed ~10% and 15% of the study area, respectively, and ~11% and ~13% of the burn area, respectively. Terrain units most likely to be influenced by changes in water level and fluvial or lacustrine erosion/deposition made up the remainder of the study area with the active and abandoned portion of the Itkillik River floodplain making up 11%, primary tundra stream gulches ~3%, and lakes ~2%.

**Figure 5:**
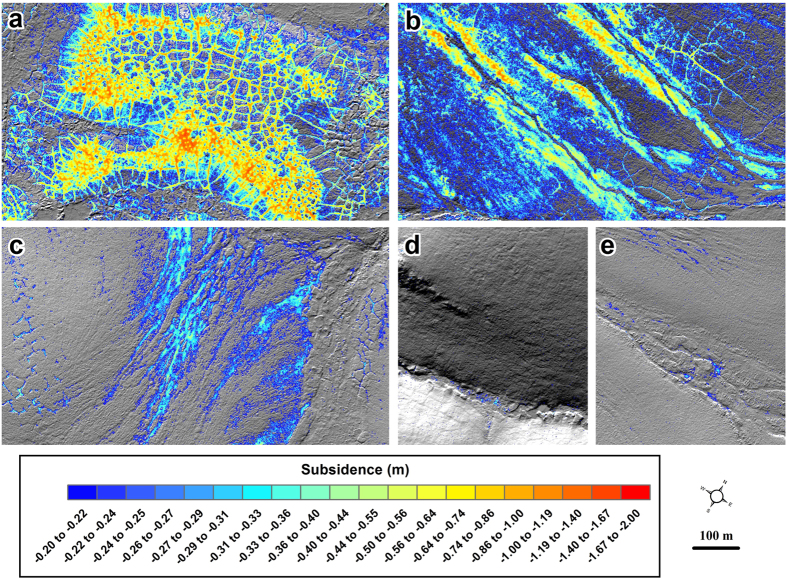
Detection of subsidence in primary upland terrain units in burned (**a–c**) and unburned tundra (**d,e**).(**a**) Widespread ice wedge degradation and thermokarst development in burned, yedoma upland terrain. (**b**) Thaw-related, hillslope feature development in burned, rocky upland terrain associated with water tracks and gully formation. (**c**) Spatially limited thaw-related landform development in burned, previously glaciated upland, primarily associated with watertracks. Unburned upland tundra sites are shown for reference with (**d**) representing a yedoma upland and stream gulch and (**e**) a previously glaciated upland. All panes show detectable subsidence based on the FIS spatially variable estimate of uncertainty and propagation of errors in the dDTM output. Figure created in Esri® ArcMap™ 10.1.

Quantitative analysis of subsidence across these terrain units revealed differences in detectable thermokarst development during the first seven years since the tundra fire (Fig. 5 and Table 1). Yedoma uplands were most heavily impacted by terrain subsidence post-fire, with measurable subsidence across ~50% of the entire terrain unit. Thaw subsidence in this terrain unit also accounted for ~46% of the total areal change detected and ~50% of the total volumetric subsidence detected in the study area (Table 1). By comparison, detected subsidence in yedoma uplands outside of the burn area was negligible (0.4%). The fact that yedoma uplands accounted for only ~30% of the burned portion of the study area but nearly 50% of the total volumetric subsidence reflects the contribution of thawing massive ice wedges. Yedoma uplands also had the highest maximum subsidence (6.7 m) values observed for the burned portion of the study area. In contrast, 45% of the rocky upland area represented detectable change, but only contributed 14% of the total volumetric subsidence between 2009 and 2014. This likely reflects not only the smaller relative contributing area of this terrain unit but also that subsidence is limited in this setting due to a thin sediment overburden and the shallow distribution of ground-ice. Previously glaciated terrain accounted for the lowest proportion of the total detectable change among upland terrain units in the burned portion of the study area (~11%) and also contributed the least to total detectable volumetric change in the burn area (~10%). Although drained lake basins contributed about the same proportion of the study area as yedoma uplands (30%), about 20% of its area showed detectable subsidence and it contributed about 20% to the total volumetric change occurring in the study period. The river floodplain and tundra stream gulches in the burn area contributed about 2% to the total detected aerial and volumetric change, whereas lakes contributed less than 1%. The only unit that contributed more volume loss outside of the burn perimeter was the floodplain area representing the active, dynamic portion of the Itkillik River.

### Increase in landscape microtopography

**Figure 6:**
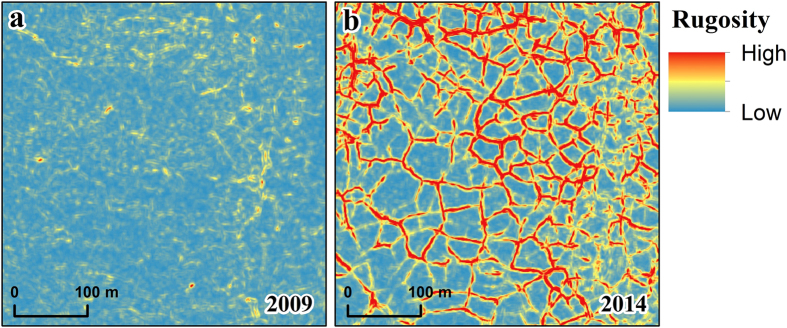
Thermokarst development has resulted in an increase in landscape-scale microtopography. Example images from 2009 (left) and 2014 (right) showing rugosity, or surface roughness, for the same location of a yedoma upland. The widespread degradation of ice wedges has increased microtopography in yedoma uplands by 340% in the aftermath of the Anaktuvuk River fire (see Supplementary Fig. S4). Figure created in Esri® ArcMap™ 10.1.

We calculated landscape rugosity for the 2009 and 2014 LiDAR datasets (see methods section) to determine potential increases in micro-topography caused by post-fire thermokarst development. Landscape rugosity more than doubled within the burn perimeter, whereas only negligible changes were measured outside of the burn area (Table 1). When analyzing rugosity changes according to the different terrain units in the study area, the burned uplands experienced the most notable increase (Fig. 6 and Table 1). Rugosity in yedoma uplands increased by ~340%, in rocky uplands by ~210%, and in glaciated uplands by ~180%. Rugosity in drained lake basins and in tundra stream gulches also increased (~80% and 30%, respectively), but to a much lesser degree. The river floodplain (−82%) and lakes (−50%) both indicated lower rugosity in the 2014 data relative to the 2009 data, which likely resulted from fewer surface waves on standing water in the study area on the latter LiDAR acquisition date. However, between 2009 and 2014 two lakes drained within the burn perimeter as a result of ice wedge degradation and migration of the Itkillik River created new river barrens. Both of these factors could account for some of the observed smoothing in the rugosity in these terrain units.

### Potential Relation Between Burn Severity and Subsidence

Stratifying the detected subsidence between 2009 and 2014 relative to the terrain units in the study area revealed different magnitudes of change relative to their contributing area within the study domain. To assess one factor that might explain this variability, we used the mean differenced normalized burn ratio (dNBR) index derived from pre- and post- fire Landsat images as calculated by Kolden and Rogen^43^ (see methods section). The three upland terrain units had the highest dNBR values, indicating greater burn severity than in the low-lying terrain units (i.e., river floodplain, tundra stream gulches, and drained lake basins). Correlating the detectable area of subsidence (% of terrain unit) with the mean dNBR across the terrain units resulted in an r^2^ of 0.83.

## Discussion

**Figure 7:**
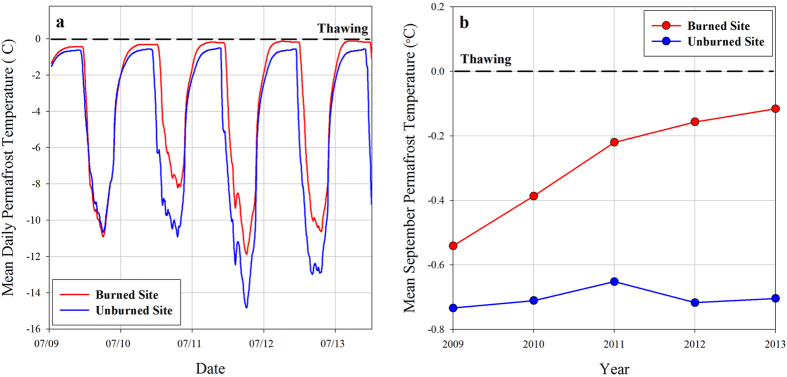
Permafrost warming in response to the tundra fire event. (**a**) Mean daily permafrost temperature data (1 m depth) recorded between July 2009 and January 2014 at a severely burned and unburned yedoma upland tundra site. (**b**) Mean September permafrost temperature (1 m depth) between 2009 and 2013. The mean annual ground temperature at the burned site has increased by 1 °C since 2009 and the mean monthly September temperature has increased from −0.5 °C to −0.1 °C since 2009. Figure created in SigmaPlot® 10.

The potential vulnerability of permafrost in the Arctic to fire-induced degradation and thermokarst development has been underestimated. Development of ALDS and RTS were observed during the first two summers following the fire^20^(Fig. 2). However, they remained limited spatially and subsequent studies concluded that the fire had little impact on the permafrost-influenced terrain^15^,^46^. In contrast, our observations show widespread ice-wedge degradation and associated terrain subsidence began after the fourth year following the tundra fire (Figs 2 and 3). Our landscape-scale perspective of thermokarst initiation following the fire, made possible by the multi-temporal airborne LiDAR datasets, indicates that 34% of the burned tundra area analyzed experienced detectable subsidence between 2009 and 2014. Yedoma uplands are the most vulnerable terrain unit to thermokarst development following tundra burning, accounting for ~50% of the detectable change in area and volume over the study period. In addition, observations of ground temperature since 2009 at a depth of 1 m in the center of an ice wedge polygon, both within (high burn severity) and outside the burn area, in the yedoma upland terrain unit indicate that thermokarst development may continue since the ground thermal regime is still re-equilibrating to the fire disturbance (Fig. 7).

Jones *et al.*^14^ hypothesized that the landscape-scale impacts of the Anaktuvuk River fire would be different across the extent of the burn area based on variability in ground-ice content at the landscape-scale^44^. In general, this appears to be the case based on the stratification of thermokarst development according to the terrain unit classification. However, analysis of the Landsat-derived burn severity index^43^ indicated a potential relation between post-fire thermokarst development and the severity with which the tundra burned that warrants further investigation (see Supplementary Fig. S3). More work is needed to separate the impacts of burn severity and landscape properties on thermokarst development.

Estimates from the large Anaktuvuk River tundra fire of 2007 suggest that vegetation and soil organic carbon combustion during the fire emitted an amount of carbon equivalent to the annual net C sink for the entire Arctic tundra biome^31^. Our observations of permafrost degradation and thermokarst development in the first seven years following the fire has also likely led to the mobilization of carbon previously frozen in permafrost^8^,^32^,^47^. In addition, our landscape scale assessment of changes in rugosity derived from the two airborne LiDAR datasets showed increases of 340% for yedoma uplands, 210% for rocky uplands, 180% for glaciated uplands, and 80% for drained lake basins. These changes in microtopography will undoubtedly factor into ongoing vegetation succession post-fire^15^ as well as impact winter snow accumulation and summer surface runoff.

Our analysis of multi-temporal airborne LiDAR acquired in 2009 and 2014 captured widespread ice-rich terrain subsidence following the Anaktuvuk River fire. However, since we lack pre-fire LiDAR data our estimates of permafrost thaw subsidence should be viewed conservatively. For example, we missed the period in which the small-scale ALDS and RTS features developed^20^ (Fig. 2). In addition, the formation of thermokarst pits due to ice wedge degradation is commonly associated with the ponding of water^48^. Any newly formed water-filled thermokarst pits in the burn area would obscure our means of detecting true subsidence in the LiDAR data, as the LiDAR we used would capture the water surface instead of the bottom surface of a pond, and thus make our results on subsidence conservative in such areas. However, visual analysis of very high-resolution satellite imagery indicates that much of the permafrost degradation occurring in the study area has not been associated with the ponding of water in ice wedge troughs (Fig. 3). In the absence of widespread ponding, we assume that the LiDAR datasets do capture the majority of thaw subsidence through July 2014. Our findings also suggest that traditional remote sensing methods based on high-resolution panchromatic imagery relying on a decrease in gray scale values from undisturbed tundra to water-filled ice wedge troughs^48^,^49^,^50^ would not adequately capture thermokarst development in response to the Anaktuvuk River tundra fire. Multi-temporal airborne LiDAR has been seldom used to quantify changes occurring in permafrost-influenced Arctic terrain^51^ but it provides a useful means of detecting the development of terrain subsidence caused by thermokarst development since it allows for a direct measure of land surface elevation relative to a geodetic reference frame. Thus, the widespread development of thermokarst in the aftermath of the Anaktuvuk River fire might have gone largely unnoticed without the acquisition and comparison of the two LiDAR datasets.

Our findings indicate that fire disturbances, projected to increase in frequency, magnitude, and severity in a warming Arctic^39^,^52^ will play a major role for permafrost degradation and associated landscape change and ecosystem shifts in tundra regions in the future. The findings presented in our study, made possible by the analysis of multi-temporal airborne LiDAR datasets, also raises several questions pertaining to thermokarst development as a result of past fires in tundra ecosystems. For example, (1) can the relation between thermokarst development and burn severity observed in our study area be used to determine permafrost thaw subsidence across the remainder of the Anaktuvuk River fire as well as other historic tundra fires, (2) what is the role of thermokarst development on snow pack accumulation and subsequent snowmelt run-off, and (3) how is vegetation succession being driven by post-fire thermokarst development? Further analysis of the novel data presented in this paper will allow us and others to further elucidate on the role of tundra fires on landscape change in the Arctic.

## Methods

### LiDAR data acquisition and processing

The 2009 airborne LiDAR dataset was acquired for Kodiak Mapping Inc. by Airborne Imaging Inc. for a road planning project managed by the Alaska Department of Transportation. This dataset encompassed 650 km^2^ of the burn area as well as adjacent unburned tundra, capturing gradients in burn severity and permafrost ground-ice content. The data were acquired between 27 June and 02 July 2009 at an estimated density of 2 points per square meter (ppm) using an Optech ALTM 3100 LiDAR system flying at an altitude of 1000 m. The vertical accuracy of this dataset was tested against two differential GPS (DGPS) field survey datasets acquired during the same month resulting in a vertical root-mean-square error (RMSE) between 0.10 m and 0.13 m. We acquired the 2014 LiDAR dataset through Kodiak Mapping Inc. under USGS contract (G10PC00057). The data were acquired between 29 July and 31 July 2014 at a nominal density of 8 ppm using a Riegl VQ 480i LiDAR system flying at an altitude of 600 m. The vertical accuracy of this dataset was tested against field survey DGPS location data acquired during the same month, which resulted in a global adjustment of 0.17 m to account for vertical bias in the data. Following the adjustment, the mean RMSE between the LiDAR data and the field survey data was 0.09 m.

Bare-earth, digital terrain models (DTMs) were created from the classified LiDAR point cloud data using the software package Quick Terrain Modeler (QTM) v. 8. Both datasets were delivered as. las files in the same projection (NAD83, UTM zone 5N) and with GEOID12A derived orthometric heights. Since the 2009 dataset was collected at a mean density of ~2.0 ppm and the 2014 dataset was collected at a mean density of ~8.0 ppm, we decimated the 2014 dataset to match the point density associated with the 2009 LiDAR dataset. This resulted in a mean ppm of 2.3 for the entire 2014 dataset after the decimation process, whereas the 2009 datasets had a mean ppm of 1.6 for the study area. Both ~2.0 ppm classified point cloud datasets were then interpolated to a 1 m grid using the last return (ground) points. The adaptive triangulation fill method, mean Z algorithm, and maximum distance to a real point of 1.0 m gridding options formed the interpolation in QTM. Both gridded datasets were then checked for concurrency, ensuring that both DTMs had the same number of columns and rows and the same bounding coordinates representing their spatial domains. Owing to the strict criteria used to create the DTM grid, a number of no data pixels occurred in each dataset. These were primarily associated with a lack of adequate returns over surface water features. Combined, these no data pixels accounted for 3.7 km^2^ of the overlapping extents of the two datasets and these missing data grid cells were subsequently masked from both DTM datasets.

### DTM Differencing

The orthogonal (1 m resolution) and concurrent (same spatial extent) 2009 and 2014 DTMs were differenced using the Geomorphic Change Detection software (v. 6) in ArcGIS^45^,^53^. Uncertainty in each DTM was determined using a three input, fuzzy inference system (FIS), spatially variable estimate of elevation uncertainty based on the vertical RMSE of each LiDAR dataset, the number of points per square meter (ppm), and the slope of the terrain. This resulted in a per pixel output of elevation uncertainty. Detectable change represented propagation of the FIS elevation uncertainties in the two datasets relative to the absolute difference in elevation for a given pixel. The dDTMs were produced using the propagation of errors threshold as well as the 95% probability threshold based on calculation of a student’s t-score^45^,^53^. Figure 4 shows the comparison between the dDTMs based on the propagation of errors and the 95% probability threshold along the northern boundary of the burn area. The images show that the propagation of errors in the dDTM resulted in detecting changes that visually appeared to have occurred between the two datasets (>~0.2 m), whereas the 95% probability threshold captured the larger magnitude changes (>~0.5 m), but underrepresented other spatially important changes.

To further gauge the quality of the datasets, four 60 × 60 m regions located outside of the burn area, representing primary terrain units in the study area, were extracted from the 2009 and 2014 DTMs (see Supplementary Fig. S1). The mean difference among the 3600 points at each site were (a) 0.02 m in an unburned, vegetated area of the floodplain, (b) −0.01 m in an unburned, drained lake basin, (c) −0.05 m in an unburned, glaciated upland, and (d) −0.06 in an unburned, yedoma upland. In all test areas the maximum difference between the two datasets was less than 0.20 m. The two upland test areas (c and d) showed a tendency towards lower elevation values in the 2014 dataset relative to the 2009 dataset. While this could represent detection of isotropic subsidence over the five-year period of our study it was below our spatially variable propagated estimate of error.

### Terrain Unit Mapping

The Land Facet Corridor Designer extension for ArcGIS^54^ was used to develop a terrain unit map for the study area based on calculation of a topographic position index (TPI) at two different scales^55^. The 2009 DTM was resampled to a spatial resolution of 30 m to capture differences in the macro-scale landscape features in the study area. The 30 m DTM was then used to create the generalized terrain unit map by calculation of a TPI grid using a standardized elevation in 750 m and 1000 m windows. The 750 m TPI was used to delineate the tundra stream gulches (TPI <= –1 standard deviation (SD)). The 1000 m TPI was used to classify the landscape into lower slopes (–1SD < TPI <= −0.5 SD). Flat slopes and middle slopes were generated with the same TPI criteria (−0.5 SD < TPI <=  0.5 SD) but distinguished by values above and below a slope of 0.5°, respectively. Upper slopes (0.5 < TPI <= 1) and ridges (TPI >1) completed the classification scheme. This five class, two-scale TPI output was then manually categorized relative to existing surficial geology maps^44^ and refined into these terrain units: (1) drained lake basins, (2) yedoma uplands, (3) rocky uplands, (4) glaciated upland, (5) river floodplain, and (6) tundra stream gulches. A seventh landform type, lakes, were extracted from a 2002 Interferometric Synthetic Aperture Radar DTM and added to the terrain unit map (see Supplementary Fig. S2).

The seven-class terrain unit data layer was used to analyze variability in subsidence across the study domain, both inside and outside the burn perimeter, using the FIS propagated error dDTM. This analysis was conducted in the Geomorphic Change Detection software^45^,^53^ using the budget segregation tool. Output from the budget segregation tool included percent of the terrain unit with detectable change and the total volumetric change in the terrain unit (Table 1).

### Surface Relief Changes

Surface relief, also known as rugosity or the ratio of surface area to planar area, was calculated for both 1m DTMs using the terrain ruggedness tool and a 5 pixel × 5 pixel moving window in the Benthic Terrain Modeler extension for ArcGIS^56^ to assess changes in microtopography post-fire. Mean rugosity values were then determined for each of the terrain units in the 2009 and 2014 DTMs and their differences reported as a percent change (Table 1).

### Burn Severity Mapping

The normalized burn ratio (NBR) index was determined from pre-fire and post-fire Landsat ETM+ (14 July 2001) and Landsat TM (14 June 2008) images, respectively^43^. A differenced NBR (dNBR) raster dataset was then created by subtracting the post-fire image from the pre-fire image^43^. The terrain units within the burn area were used to calculate a mean dNBR value (see Supplementary Fig. S2). These mean terrain unit dNBR values were then related to detectable subsidence occurring in the burned portion of the study area (see Supplementary Fig. S3).

### Very high-resolution satellite imagery

Very high-resolution satellite imagery from the DigitalGlobe^TM^ constellation of satellites was acquired over select locations inside and outside of the burn perimeter. Images were acquired opportunistically, both spatially and temporally, and were used for visual corroboration of changes detected in the airborne LiDAR data.

### Permafrost Temperature Observations

We installed a shallow (1 m depth) permafrost temperature data logger (Hobo U23 Pro v2) at a high burn severity, yedoma upland tundra site, and an unburned, yedoma upland tundra site in July 2009. The data logger was configured to record a temperature measurement every hour. Data from the two data loggers were summed to mean daily and mean monthly temperature records for both sites and provide an indication of the processes associated with the widespread thermokarst development observed in the multi-temporal airborne LiDAR datasets.

## Additional Information

**How to cite this article**: Jones, B. M. *et al.* Recent Arctic tundra fire initiates widespread thermokarst development. *Sci. Rep.* **5**, 15865; doi: 10.1038/srep15865 (2015).

## Supplementary Material

Supplementary Information

## Figures and Tables

**Figure 1:**
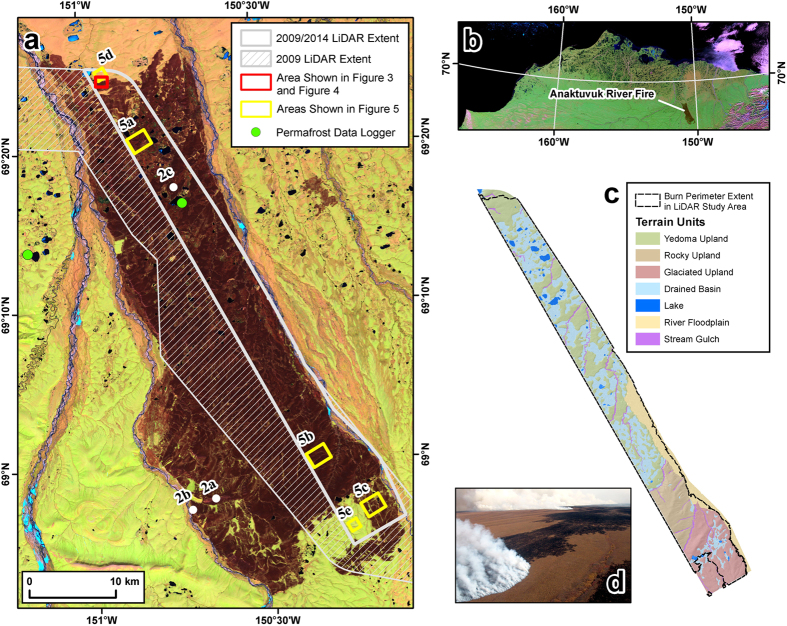
The Anaktuvuk River tundra fire study area. (**a**) Landsat-5 TM image acquired on 14 June 2008, the first summer after the Anaktuvuk River tundra fire, showing the ~1,000 km^2^ burn area. The extent of the 2009 airborne LiDAR dataset is shown with a hatched grey line and the overlapping extent of the 2009 and 2014 airborne LiDAR datasets are outlined with the bold grey line. Various sites mentioned in the text and shown in other figures are marked accordingly. (**b**) Inset map showing a MODIS satellite image of northern Alaska and the location of the Anaktuvuk River fire burn area. (**c**) The terrain unit map created for the multi-temporal airborne LiDAR study area. The extent of the burn within this area is denoted with the dashed black line. (**d**) An oblique aerial photograph from early September 2007 during a period of severe and widespread burning (photo credit: CDA). Map created in Esri® ArcMap™ 10.1.

**Table 1:** 

Terrain Units	Terrain Unit Area#	Detected subsidence derived from multitemporal airborne LiDAR data*					Change in Microtopography^	Burn Severity♮
		Area		Volume		Max. Sub.		
	(km^2^)	(km^2^)	(% of terrain unit)	×10,000 (m^3^)	(% of total)	(m)	(%)	(dNBR)
Drained Lake Basin								
Inside Burn	98.2	22.88	23.29	644.6	19.34	3.9	81.3	590
Outside Burn	2.4	0.03	1.35	0.9	0.03	3.3	−4.4	—
Yedoma Upland								
Inside Burn	98.6	48.61	49.31	1653.6	49.62	6.7	343.7	753
Outside Burn	4.1	0.02	0.40	0.7	0.02	3.2	−5.8	—
Rocky Upland								
Inside Burn	34.5	15.40	44.63	468.7	14.07	3.3	211.0	782
Outside Burn	—	—	—	—	—	—	—	—
Glaciated Upland								
Inside Burn	39.2	11.18	28.53	340.4	10.21	3.1	178.0	675
Outside Burn	10.8	0.05	0.51	1.3	0.04	3.3	−0.9	—
River Floodplain								
Inside Burn	20.2	2.18	10.79	59.9	1.80	3.0	−82.1	609
Outside Burn	16.5	1.60	9.70	71.8	2.16	4.1	7.4	—
Tundra Stream Gulch								
Inside Burn	10.0	2.39	23.96	71.0	2.13	5.7	30.1	634
Outside Burn	1.0	0.04	3.55	1.0	0.03	1.9	8.6	—
Lakes								
Inside Burn	5.6	0.69	12.28	18.1	0.54	2.9	−49.6	—
Outside Burn	0.4	0.02	4.68	0.5	0.02	1.0	−55.6	—
Totals								
Inside Burn	306.4	103.3	—	3256.3	—	6.7	101.8	—
Outside Burn	35.2	1.8	—	76.2	—	4.1	−8.4	—

Table 1. Detected subsidence in the multi-temporal airborne LiDAR datasets. Change between the 2009 and 2014 datasets determined using an FIS uncertainty analysis and the propagation of errors on a per pixel basis^45^,^53^. Subsidence is reported in terms of area affected and volumetric lowering by terrain unit and whether the area was inside or outside of the burn perimeter. Change in microtopography was determined using a rugosity metric^56^ and the Landsat-derived burn severity index is from Kolden and Rogan^43^.

^#^Based on terrain unit classification developed for the study area (see methods section) and existing surficial geology maps^44^.

^*^Detected changes in the dDTM based on the FIS analysis and propagation of the elevation uncertainties in the dDTM calculation^45^,^53^ using the 2009 and 2014 airborne LiDAR datasets.

^^^Based on changes in rugosity between the 2009 and 2014 datasets (see methods section).

^♮^Mean differenced normalized burn ratio (dNBR) derived from pre- and post-fire Landsat image pairs^43^.
