# Supplementary material for: Recent Arctic tundra fire initiates widespread thermokarst development
Source: Sci Rep. 2015 Oct 29;5:15865. doi: 10.1038/srep15865 (PMC4625366; doi:10.1038/srep15865)
Supplement: Supplementary Information [file srep15865-s1.pdf]

Recent Arctic tundra fire initiates widespread thermokarst development

Benjamin M. Jones[1*], Guido Grosse[2], Christopher D. Arp[3], Eric Miller[4], Lin Liu[5], Daniel J. Hayes[6] & Christopher F. Larsen[7]

[1]Alaska Science Center, U.S. Geological Survey, Anchorage, AK 99508, USA,

[2]Alfred Wegener Institute, Helmholtz Centre for Polar and Marine Research, Potsdam, Germany,

[3]Water and Environmental Research Center, University of Alaska Fairbanks, Fairbanks, AK 99775, USA,

[4]Bureau of Land Management Alaska Fire Service, Fairbanks, AK 99709, USA,

[5]Earth System Science Programme, Faculty of Science, Chinese University of Hong Kong, Hong Kong, China,

[6]Environmental Sciences Division, Oak Ridge National Laboratory, Oak Ridge, TN 37831 USA,

[7]Geophysical Institute, University of Alaska Fairbanks, Fairbanks, AK 99775 USA

*Correspondence and requests for material should be addressed to B.M.J. (bjones@usgs.gov)

Supplementary Information Files

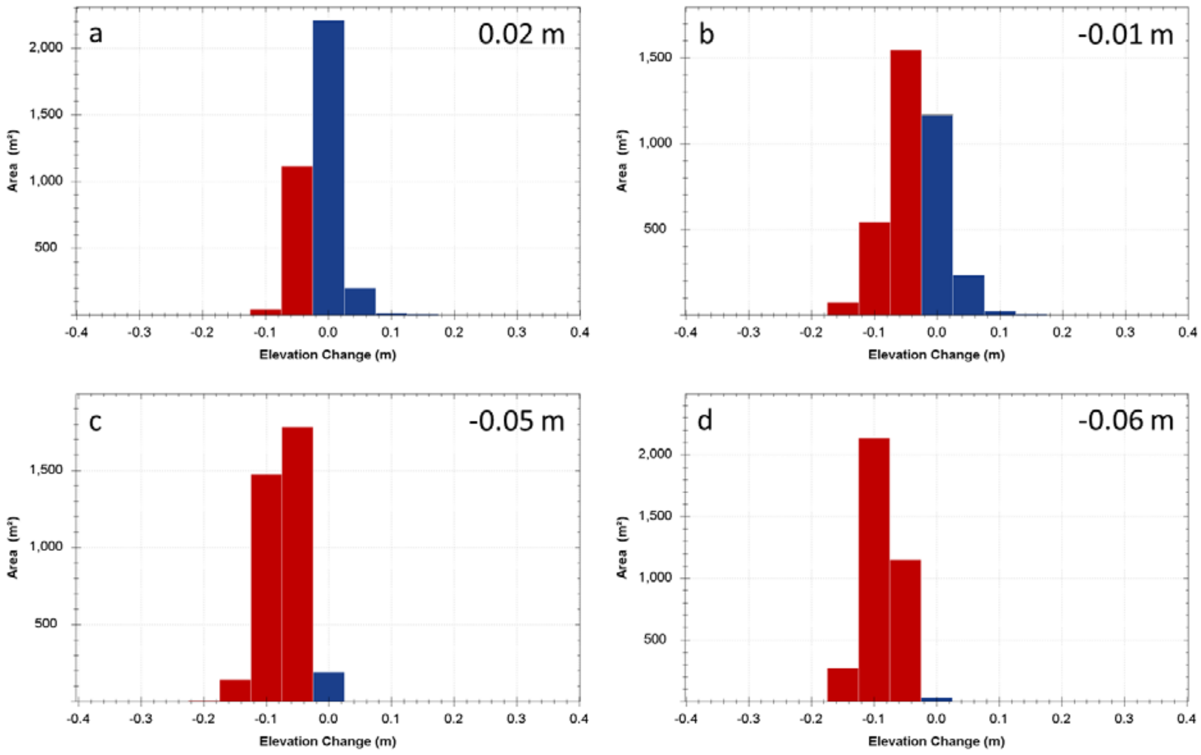

Supplementary Figure S1. Comparison of the two LiDAR datasets in unburned tundra settings. Four 60 x 60 m regions, representing primary landforms in the study area, located outside of the burn area were extracted from the 2009 and 2014 DTMs to gauge the quality of the datasets. Red represents values lower in the 2014 dataset and blue represents values higher in 2014 dataset. The mean difference among the 3600 points at each site were (a) 0.02 m in an unburned, vegetated area of the floodplain, (b) -0.01 m in an unburned, drained lake basin, (c) -0.05 m in an unburned, glaciated upland, and (d) -0.06 in an unburned, yedoma upland. In all test areas the maximum difference between the two datasets was less than 0.18 m. The two upland test areas (c and d) showed a tendency towards lower elevation values in the 2014 dataset relative to the 2009 dataset. While this could represent detection of isotropic subsidence over the five year period of our study it was below our fuzzy inference system spatially variable propagated estimate of error. Figure created in SigmaPlot® 10.

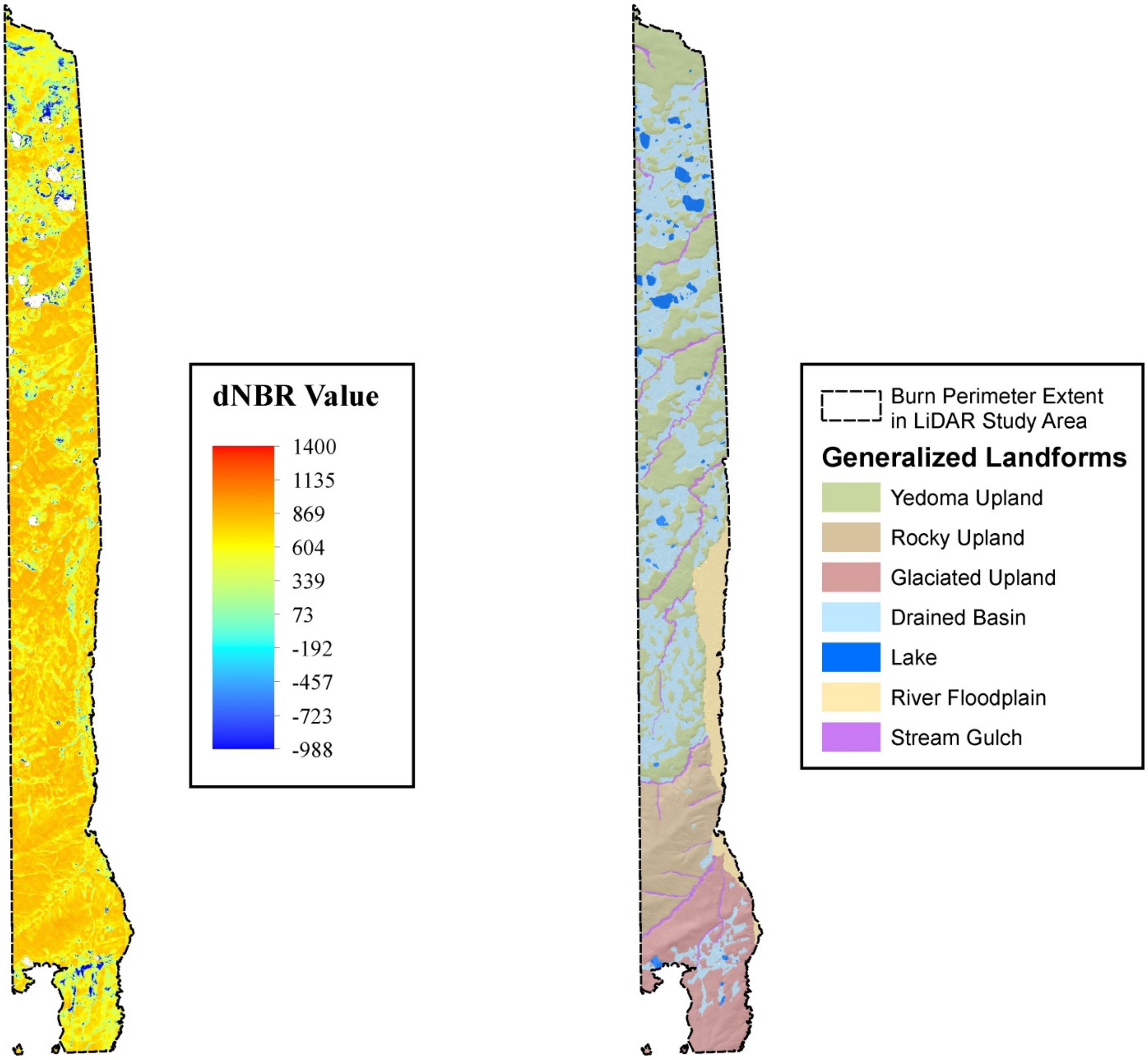

Supplementary Figure S2. (a) Landsat-derived burn severity and (b) the terrain unit map created for the study area. The burn severity map is from Kolden and Rogen[43] and the terrain unit map was based on methods developed by Weiss[55] and manual interpretation. Lakes are masked as white (no data) in (a). Figure created in Esri® ArcMap™ 10.1.

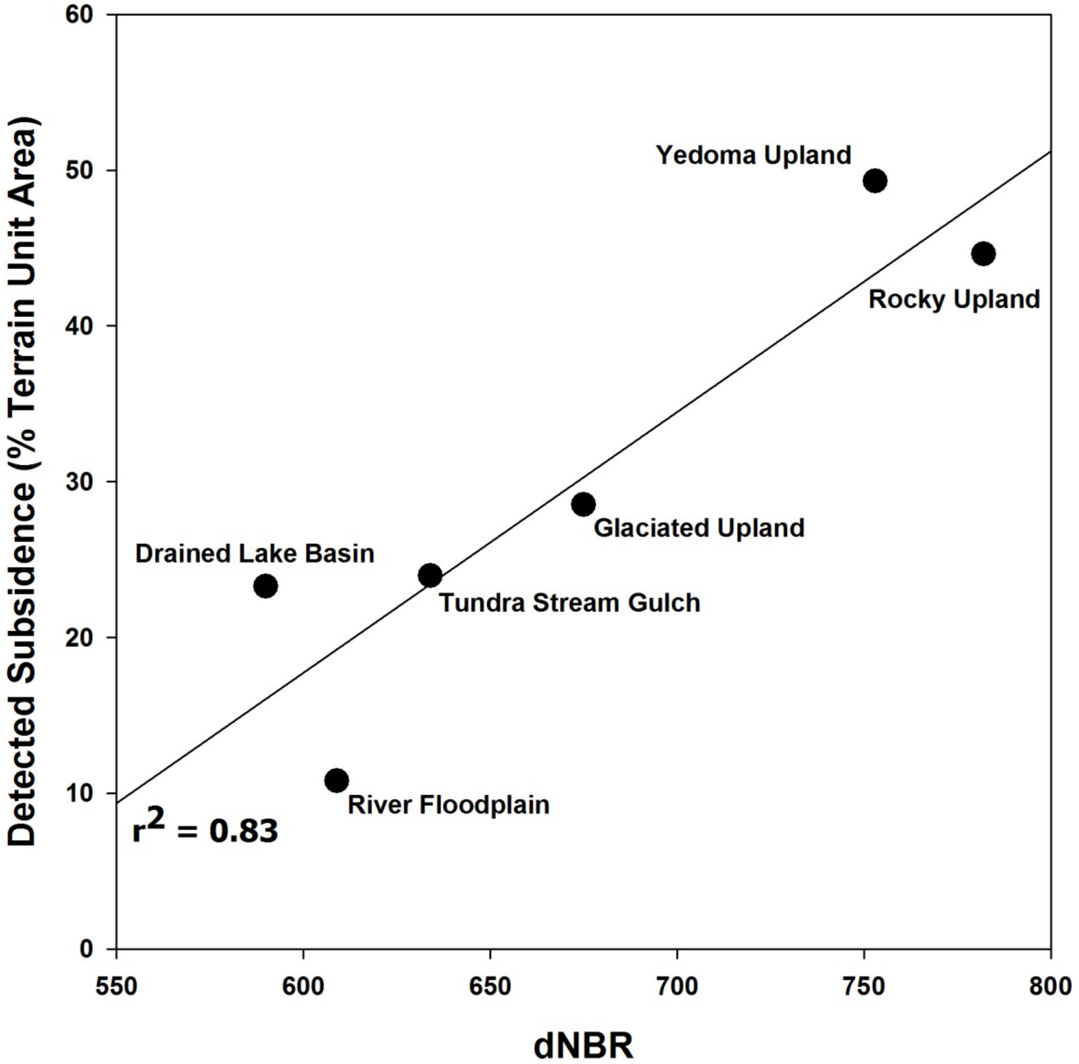

Supplementary Figure S3. Positive relation between burn severity and thermokarst development. dNBR data from Kolden and Rogen[43] compared with detected subsidence for the six vegetated terrain units reported as a percent of the terrain unit area impacted. Figure created in SigmaPlot® 10.

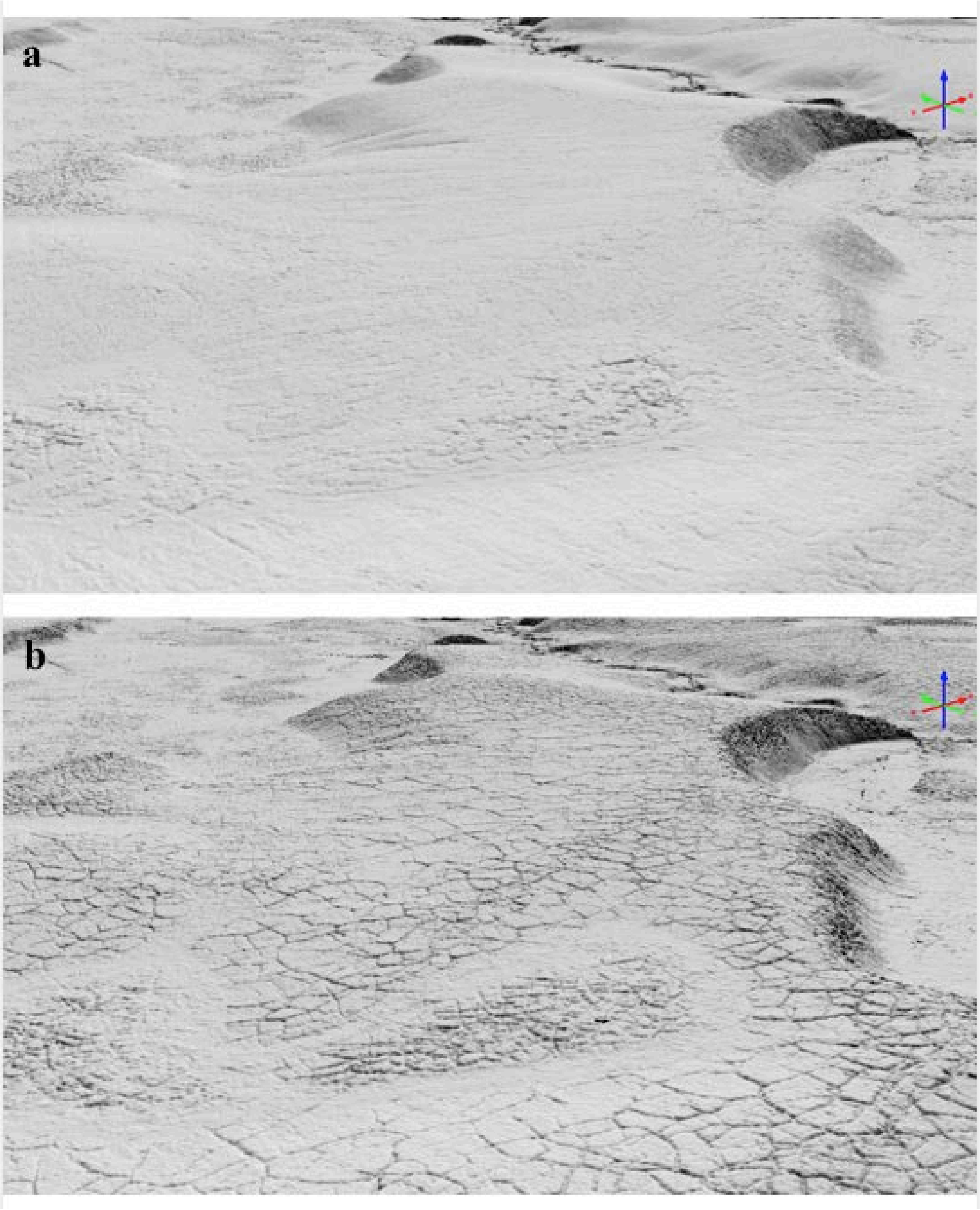

Supplementary Figure S4. Increase in surface roughness triggered by thermokarst development. Perspective hillshade images of the (a) 2009 and (b) 2014 LiDAR DTMs peering over a yedoma upland. Both images are shown with a 3x vertical exaggeration. Figure created in Esri® ArcScene™ 10.1.
